# Epigenetic Activation of Silent Biosynthetic Gene Clusters in Endophytic Fungi Using Small Molecular Modifiers

**DOI:** 10.3389/fmicb.2022.815008

**Published:** 2022-02-14

**Authors:** Lynise C. Pillay, Lucpah Nekati, Phuti J. Makhwitine, Sizwe I. Ndlovu

**Affiliations:** Discipline of Medical Microbiology, School of Laboratory Medicine and Medical Sciences, College of Health Sciences, University of KwaZulu-Natal, Durban, South Africa

**Keywords:** endophytic fungi, biosynthetic gene cluster, histone deacetylases, epigenetic modifiers, chromatin network, secondary metabolites

## Abstract

The discovery of silent biosynthetic gene clusters (BGCs) in fungi provides unlimited prospects to harness the secondary metabolites encoded by gene clusters for various applications, including pharmaceuticals. Amplifying these prospects is the new interest in exploring fungi living in the extremes, such as those associated with plants (fungal endophytes). Fungal species in endosymbiosis relationship with plants are recognized as the future factories of clinically relevant agents since discovering that they can produce similar metabolites as their plant host. The endophytes produce these compounds in natural environments as a defense mechanism against pathogens that infect the plant host or as a strategy for mitigating competitors. The signaling cascades leading to the expression of silent biosynthetic gene clusters in the natural environment remain unknown. Lack of knowledge on regulatory circuits of biosynthetic gene clusters limits the ability to exploit them in the laboratory. They are often silent and require tailor-designed strategies for activation. Epigenetic modification using small molecular compounds that alter the chromatin network, leading to the changes in secondary metabolites profile, has achieved considerable success. This review aims to comprehensively analyze the secondary metabolite profiles expressed after treatment with various epigenetic modifiers. We first describe the regulatory circuits governing the expression of secondary metabolites in fungi. Following this, we provide a detailed review of the small molecular modifiers, their mechanism(s) of action, and the diverse chemistries resulting from epigenetic modification. We further show that genetic deletion or epigenetic inhibition of histone deacetylases does not always lead to the overexpression or induction of silent secondary metabolites. Instead, the response is more complex and often leads to differential expression of secondary metabolites. Finally, we propose using this strategy as an initial screening tool to dereplicate promising fungal species.

## Introduction

Endophytic fungi have recently attracted attention in screening programs after discovering that they can produce similar metabolites to their plant hosts (Deepika et al., 2016). Endophytic microorganisms colonize the intracellular and intercellular regions of healthy plant tissues without damaging the host plant (Rodriguez et al., 2009; Nair and Padmavathy, 2014). In this relationship, they produce secondary metabolites that play an essential role in defending the plant host from pathogens, thus protecting its habitat (Hardoim et al., 2015; Deepika et al., 2016). Secondary metabolism is not directly involved in the producing organism’s growth, development, or reproduction. Still, it may act as chemical signals during cell-to-cell communications in natural environments (Netzker et al., 2015). Interestingly, the produced secondary metabolites exhibit sought-after properties in the pharmaceutical industries such as antibacterial, antifungal, anticancer, and other bioactivities (Tyurin et al., 2018).

Secondary metabolites (SMs) are low molecular weight substances encoded by contiguous gene assembles termed biosynthetic gene clusters (BGCs), which encode enzymes responsible for a stepwise assembly of complex bioactive molecules (Pfannenstiel and Keller, 2019). Many of the fungal-derived secondary metabolites, such as cyclosporines, statins, and penicillins were discovered in the early drug screening programs (Harvey et al., 2018; Ding et al., 2020). However, the search for active molecules from microorganisms declined due to a continuous rediscovery of known metabolites (Jackson et al., 2018). As a result, pharmaceutical companies shifted their investment interests to synthetic drug development routes (Jackson et al., 2018).

Recent advances in genome sequencing technologies have revealed that fungi are host to many novel BGCs other than those previously identified in natural product screening programs (Macheleidt et al., 2016). Fungi, more especially those from unexplored environments such as fungal endophytes, are promising alternative sources of novel chemistries that will provide potent drugs that have the potential to combat the growing challenge of antibiotic resistance and the emergence of new multidrug-resistant pathogens (Phukan et al., 2018). However, the problem is that most of these BGCs do not produce appreciable concentrations of metabolites during cultivation in standard laboratory conditions and are regarded as “silent” or “cryptic” (Xu et al., 2017; Pfannenstiel and Keller, 2019). Therefore, there is a discrepancy between fungi’ secondary metabolite production potential and the currently known fungal metabolites in the clinical pipeline. For this reason, there is a fast-growing research interest aimed at identifying new approaches to activating the silent secondary metabolites encoding genes (Macheleidt et al., 2016).

Successful activation of silent BGCs requires a proper understanding of the regulatory circuits governing the secondary metabolites gene clusters. The signaling cues that trigger the expression of the silent or transiently expressed BGCs in natural environments remain unknown (Reen et al., 2015). Several strategies developed to awaken the ‘silent’ genes have shown to significantly influence the profile of secondary metabolites produced (VanderMolen et al., 2013; Hewage et al., 2014; Nielsen and Larsen, 2015; Begani et al., 2018; Scherlach and Hertweck, 2021). The activation of these genes can be achieved at the genome, transcriptome, proteome, or metabolome levels (Scherlach and Hertweck, 2009; Cichewicz, 2010; Ochi and Hosaka, 2013; Begani et al., 2018; Scherlach and Hertweck, 2021). There are several exciting reviews on these strategies, such as those noted by Rutledge and Challis (2015), which categorizes these approaches into pleiotropic and pathway-specific.

Several BGC awakening strategies (based on culturing techniques and genetic manipulation) proposed to date have varying advantages and disadvantages. The culture-based strategies include the variation of growth conditions, also known as one strain many compounds (OSMAC) and co-culturing (Pan et al., 2019). In the OSMAC strategy, growth conditions such as temperature, media, cultivation time, etc., can be systematically varied to simulate the changing signaling cues in the environment (Bode et al., 2002; Guzmán-Chávez et al., 2018). Different studies have shown that small changes in cultivation conditions and media composition (OSMAC approach) completely shift the metabolic profile of various filamentous fungi (Bode et al., 2002; Romano et al., 2018; González-Menéndez et al., 2019; Toghueo et al., 2020). The second culture-based approach involves co-culturing with microorganisms such as bacteria, where crosstalk between the co-cultivated organisms induces the production of metabolites encoded by the silent BGCs (Bertrand et al., 2014; Rutledge and Challis, 2015; Pfannenstiel and Keller, 2019). The challenge with the culture-based approaches is that they are laborious, requiring scientists to set up several experiments encompassing different conditions (Romano et al., 2018).

Several genetic manipulation strategies have been developed to awaken the silent biosynthetic gene clusters in fungi (Jiang et al., 2021). One of these strategies involves engineering the transcription and translation machinery, which is achieved by introducing mutational changes in RNA polymerase and proteins, thus increasing BGC expression (Rutledge and Challis, 2015; Pfannenstiel and Keller, 2019). Another genetic approach includes manipulating global regulators to activate more than one pathway to discover multiple metabolites (Rutledge and Challis, 2015; Baral et al., 2018). In a review by Rutledge and Challis (2015), they discussed the four pathway-specific approaches, including (1) Manipulating pathway-specific regulators to increase the expression of BGCs. This approach requires prior knowledge of genes that encode putative pathway-specific transcription factors (Rutledge and Challis, 2015). (2) Reporter-guided mutant selection method allows for the visualization of mutant expressing a target BGC (Rutledge and Challis, 2015). (3) Refactoring by using constitutive or readily inducible promoters, replacing natural promoters (Rutledge and Challis, 2015; Fan et al., 2017; Baral et al., 2018). (4) After the BGC of interest has been identified, the genes are activated and expressed in a heterologous host. The heterologous expression approach simplifies metabolite identification workflows (Rutledge and Challis, 2015; Fan et al., 2017; Harvey et al., 2018). Refactoring is seen as the best approach since fungi are typically resistant to genetic manipulation, and heterologous expression might provide a better alternative for expressing fungal biosynthetic genes (Keller, 2019). However, there are limited successes achieved through these approaches. Although these genetic approaches have shown successes in different fungal species, their wide application in fungi remains limited. The limitations are due to fungi’s complex genetic system and the lack of genetic tools such as selection markers for application in filamentous fungi (Jiang et al., 2021). It appears that non-genetic strategies are still valuable, especially for novel fungal isolates with no previous genetic information.

Application of small molecular compounds that epigenetically modifies the chromatin leading to the induction of silent fungal BGCs have shown considerable successes (Strauss and Reyes-Dominguez, 2011; Akone et al., 2016). Despite the attention given to this approach, there are a few reviews (Cichewicz, 2010; Pettit, 2011; Poças-Fonseca et al., 2020; Toghueo et al., 2020) that consolidate the findings thus far. This review aims to analyze the successes achieved using this strategy thus far. This analysis will be achieved by exploring the secondary metabolite profiles expressed after treatment with various epigenetic modifiers. We first describe the regulatory circuits governing the expression of secondary metabolites in fungi. The relationship between the plants and fungal endophytes is presented as an example of an environmental niche with dynamic interactions and a high potential for novel chemistries. Following this, we provide a detailed review of the small molecular modifiers, their mechanism(s) of action, and the diverse chemistries resulting from epigenetic modification. We further show that genetic deletion or epigenetic inhibition of histone deacetylases does not always lead to the overexpression or induction of silent secondary metabolites. Instead, the response is more complex and often leads to differential expression of secondary metabolites. Finally, we propose using this strategy as an initial screening tool to dereplicate promising fungal species.

## Endosymbiotic Relationship Between Fungal Endosymbionts and their Plant Hosts

Endophytic fungi belong to mitosporic and meiosporic ascomycetes that asymptomatically colonize the internal tissues of the plant host (Jia et al., 2016). Endophytic fungi have a significant biological diversity, as most plants host one or more endophytic fungus species (Jia et al., 2016; Rana et al., 2019). The host plant secretes compounds as a defense system to protect itself from colonization (Jia et al., 2016). In response, the endophytic fungi secrete the matching detoxifying enzymes such as cellulases, lactase, xylanase, and protease, to decompose these plant protective compounds, thus allowing these endophytes to penetrate through the plant defense systems (Jia et al., 2016; Khare et al., 2018). Once the endophytic fungi colonize the inner tissues of the plant, fungi assume a latent state either for the whole lifetime of the plant host (neutralism) or for an extended period (mutualism or antagonism) (Jia et al., 2016).

There are several benefits for the plant host resulting from this symbiotic relationship with fungal endophytes. The benefits include induced plant growth, increased resistance to disease, and the stimulation of secondary metabolites production (Alvin et al., 2014; Fadiji and Babalola, 2020). Fungal endophytes produce secondary metabolites in response to external stimuli such as changes in nutritional needs or pathogenic threats (Alvin et al., 2014; Macheleidt et al., 2016). These secondary metabolites may serve as defense molecules mediating nutrient acquisition and species-species communication (Strobel, 2003; Berg and Hallmann, 2006; Macheleidt et al., 2016). Secondary metabolites produced by fungal endophytes can trigger a defense mechanism by the plant host against specific plant pathogens (Kloepper and Ryu, 2006; Alvin et al., 2014). Also, fungal endophytes can encourage increased cell apoptosis when pathogens infect the plant host. This response can occur in several ways, such as producing phytohormones, siderophores, etc. (Berg and Hallmann, 2006; Alvin et al., 2014). Other benefits include a natural resistance to soil contaminants, the ability to degrade xenobiotics, and their action as vectors to present degradative traits to plants (Ryan et al., 2008). The host plant also provides many benefits to the development and survival of the endophytic fungi (Fadiji and Babalola, 2020).

The host plant’s genetic background determines its endophyte population structure as substrates produced by the host plant impact the colonization and distribution of the endophytic fungi (Card et al., 2016; Jia et al., 2016). These factors indicate that the host plant’s fitness affects the fitness of endophytic fungi (Jia et al., 2016). The symbiotic relationship between the plant and its endophytes proves that endophytic bioactive compounds are likely to possess reduced cell toxicity since the secondary metabolites produced during this interaction do not harm the eukaryotic host system (Jia et al., 2016; Fadiji and Babalola, 2020). This suggests that active secondary metabolites produced by endophytic fungi in this symbiotic relationship are potentially selectively toxic to invading agents but not their plant host. Selective toxicity is an important criterion when developing pharmaceutical drugs, as potential drugs may not adversely affect recipient cells (Strobel, 2003; Alvin et al., 2014).

## Biosynthetic Gene Clusters in Fungal Species and their Regulation

### Organization of the Biosynthetic Gene Clusters in Fungal Species

Secondary metabolites are produced by contiguous gene assembles termed biosynthetic gene clusters (BGCs), which encode enzymes responsible for a stepwise assembly of complex bioactive molecules (Xu et al., 2017; Tyurin et al., 2018). The clustering of the biosynthetic genes is suggested to ensure coordinated regulation of secondary metabolism (Shwab et al., 2007). Fungal BGCs characteristically exhibit modularity of enzymatic domains. They can exceed 100 kb in size, a feature that creates bottlenecks when using molecular biology techniques to capture their activities through heterologous expression (Reen et al., 2015). Classes of BGCs include non-ribosomal peptides (NRPs), polyketides (PKs), ribosomally synthesized and post-translationally modified peptides (RiPPs), terpenoids, saccharides, and hybrid compounds being the most widely characterized (Alvin et al., 2014; Reen et al., 2015; Macheleidt et al., 2016). The NRPs are characterized by three main domains, (1) the adenylation (A) domain responsible for amino acid recognition, (2) the peptidyl carrier protein (PCP) responsible for activation as well as (3) a condensation (C) domain responsible for bond formation. Typical PKs also consists of three domains which include (1) an acyltransferase (AT) which acts as the gatekeeper for substrate specificity, selecting and activating the monomers and the intermediate acyl chain, (2) a ketosynthase (KS) which catalyzes C-C bond formation *via* the Claisen condensation in the elongation of the polyketide chain, and (3) the thioesterase (TE) domain which is responsible for the termination of the growing chain The structural details of the fungal biosynthetic gene clusters will not be covered in details in this review, but the reader is referred to an excellent review by Guzmán-Chávez et al. (2018). The fungal BGCs represent a potential source for new scaffolds to discover novel antimicrobial compounds (Tyurin et al., 2018).

Fungal biosynthetic genes tend to position proximal to the telomeres in the genome in heterochromatin regions (Brosch et al., 2008). Biosynthetic genes are often transcriptionally controlled by epigenetic regulation through histone deacetylation and DNA methylation (Pettit, 2011). The changes in biotic and abiotic conditions in the natural environments trigger the fungal chromatin-based induction of silent secondary metabolites biosynthetic gene clusters. Essentially, these triggers are environmental factors and nutrients which strongly influence the production of secondary metabolites by fungi (Zutz et al., 2013). However, fungal fermentations on artificially defined media in the laboratory tend to be poor surrogates to mimic the endophytic environmental conditions. This often results in the induction of only a subset of biosynthetic pathways encoding secondary metabolites thus limiting the full biosynthetic prospects of fungi (Williams et al., 2008). Understanding the regulatory processes will assist in realizing the full potential of endophytic fungi.

## Genetic Regulation of Fungal Biosynthetic Gene Clusters Encoding Secondary Metabolite at a Chromatin Level

Secondary metabolism is not immediately essential for the organism but produced secondary metabolites may influence the organism’s competitiveness in natural environments, hence the tight regulation of these biosynthetic genes (Gacek and Strauss, 2012). Biosynthetic gene clusters encoding secondary metabolites are typically activated in response to environmental stimuli, and this expression depends on the developmental stage of the fungus (Keller, 2019). In fungi, the chromatin structure has emerged as a fundamental regulatory circuit of cellular metabolism, with the chromatin-modifying enzymes playing an essential role in fungal secondary metabolism (Brosch et al., 2008; Nützmann et al., 2013).

### The Structure of the Chromatin and Its Role in the Control of Gene Expression

The chromatin is composed of DNA (∼147 base pairs of double-stranded DNA) cross-linked to an octamer of histone proteins, H2A, H2B, H3, and H4 (Aghcheh and Kubicek, 2015; Sun et al., 2021). The tightly packed DNA and structural nuclear proteins like histones and non-histone chromatin-associated proteins together form a nucleosome (Nützmann et al., 2013). Linker histones, mainly H1, bind to the nucleosome at the entry and exit site. It does not make up the histone’ “’bead” but sits on top of the structure, which assists the chromatin in folding into higher-order structures (Fyodorov et al., 2018).

The chromatin acts as a natural substrate for the transcriptional machinery and regulatory proteins that replicate and repair DNA and chromosome segregation (Gacek and Strauss, 2012; Nützmann et al., 2013). Histones H2A, H2B, H3, and H4 exist as dimers and are the four core proteins (Fyodorov et al., 2018; Sun et al., 2021). They have a basic N-terminal domain and a histone-fold C-terminal domain (Caterino and Hayes, 2011). Histones H3 and H4 are almost identical in plants and animals, while H2A and H2B histones differ from species to species (Fyodorov et al., 2018). Histone H2A is well characterized and is found at sites with specific transcriptional control, chromatin remodeling, and when DNA repair is required (Giaimo et al., 2019). The H3 and H4 histones have long tails extending from the nucleosome, representing sites where pathways for signal transduction affect the chromatin structure. Moreover, numerous other minor histone variants play a specialized role in regulating the chromatin (Martire and Banaszynski, 2020).

**FIGURE 1:**
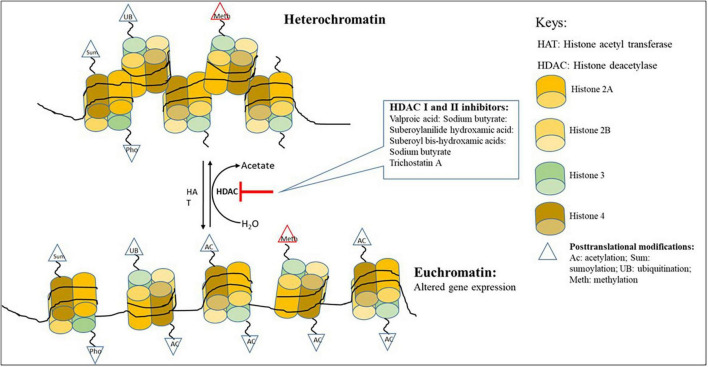
Chromatin remodeling showing the changing of heterochromatin to euchromatin with the addition of HDAC inhibitors.

Histones are substrates for several post-translational modifications (PTMs), including methylation, acetylation, phosphorylation, ubiquitination, SUMOylation, citrullination, and ADP-ribosylation that can modify their interaction with nuclear proteins and DNA (Bannister and Kouzarides, 2011; Aghcheh and Kubicek, 2015). Post-translational modifications are central in defining the state of the chromatin, which can either be loosely (euchromatin) or tightly (heterochromatin) packaged (Figure 1; Studt et al., 2013). These modifications are likely to control the interaction of histones with transcriptional activators and repressors (Shwab et al., 2007). Histone acetylation and DNA methylation are the most commonly occurring of all post-translational modifications (Studt et al., 2013). When the histones are highly acetylated, they are loosely bound to DNA and renders the chromatin transcriptionally accessible (Okada and Seyedsayamdost, 2017). However, it has been shown that the post-translational modification of histones through either methylation, acetylation and/or phosphorylation can lead to chromatin rearrangements that can improve selective accessibility of DNA by some transcription factors (Bannister and Kouzarides, 2011; Fischer et al., 2016).

Changes in the chromatin structure are essential in the functioning of the cell and may lead to short and long alterations in the transcriptional activities of the cell. These changes are governed by intrinsic cellular and extrinsic environmental factors (Brosch et al., 2008). They play a central role in maintaining cellular integrity, differentiation, development, and metabolism (Brosch et al., 2008). The intrinsic cellular factors are mediated by enzymes such as histone deacetylases (HDACs) and histone acetyltransferases (HATs) which are responsible for eliminating, establishing, and preserving local chromatin modification that either expresses or represses gene transcription (Albright et al., 2015). Histone binding proteins, histone acetyltransferase, and methyltransferases can regulate the expression of secondary metabolite BGCs in filamentous fungi (Gacek and Strauss, 2012). Two main groups of these small-molecule epigenetic modifiers: HDAC inhibitors and DNMT inhibitors, play an influential role as elicitors in producing secondary metabolites (Okada and Seyedsayamdost, 2017). The HATs and HDACs have antagonistic action on histone acetylation. HATs are responsible for acetylation, which is linked to gene transcription, and HDACs involved in deacetylation is associated with gene silencing (Bannister and Kouzarides, 2011; Studt et al., 2013).

Histone deacetylases (HDACs) are categorized into three families (1) zinc-dependent (classical) HDACs (e.g., class 1, class 2, HOS3-like HDACs in fungi, and class 4 found in other Eukaryotes), (2) nicotinamide adenine dinucleotide (NAD^+^)-dependent SIR-like HDACs (Sirtuins), and (3) HD2-like enzymes (found exclusively in plants) (Brosch et al., 2008; Lamoth et al., 2015). Classical HDACs are particularly interesting as they play a pivotal role in the regulation of secondary metabolism. Classical HDACs can be further classified into class1 or RPD3-type HDACs (RPD3; RPDA in *A. nidulans*, Hos1, Hos2; HosA in *A. nidulans*) and class 2 or HDA1-type (HDA1; HdaA in *A. nidulans*) (Tribus et al., 2010; Lamoth et al., 2015). Deleting deacetylases or chemical inhibition of deacetylase using small molecular elicitors that inactivate deacetylases’ activity often leads to increased production of known or novel secondary metabolites in several fungi (Gacek and Strauss, 2012). The HDACs and HATs are instrumental in transitioning between heterochromatin and euchromatin (Fischer et al., 2016).

### Global Regulation of Biosynthetic Gene Clusters in Fungal Species

The discovery of LaeA (loss of *aflR* expression) in *Aspergillus* species, a nuclear methyltransferase protein, has provided insights into regulating secondary metabolite production in filamentous fungi (Bok and Keller, 2004). Sequence analysis of a gene encoding LaeA protein showed that this protein is similar to histone and arginine methyltransferases (Chiang et al., 2009). Furthermore, in a study by Perrin et al. (2007), they compared the transcriptional profile of the wild type, Δ*laeA* mutant, and complemented strains of *A. fumigatus*. They showed that *laeA* was involved in the transcriptional control of 13 of the 22 secondary metabolite gene clusters of this species. Seven of these gene clusters controlled by *laeA* were located in subtelomeric regions. The location of *laeA* regulated genes and its sequence similarity to methyltransferases provided strong evidence that *laeA* might be involved in chromatin remodeling (Fox and Howlett, 2008). Apart from regulating secondary metabolite gene clusters in fungi, LaeA is also a critical factor in fungal morphology and development (Calvo et al., 2002; Bok and Keller, 2004). Global manipulation of transcription factors allows for simultaneous targeting of several secondary metabolite gene clusters (Bok and Keller, 2004). This approach has been demonstrated in *Aspergillus* spp. through the overexpression of *laeA*, which activated or enhanced the production of several known bioactive compounds (Bok and Keller, 2004). Furthermore, LaeA, alongside the highly conserved velvet complex proteins, is believed to play a role in fungal pathogenicity (Fischer et al., 2016).

The LaeA/LAE1 protein is directly involved in the transcription of various secondary metabolite gene clusters through direct interaction with the velvet domain family (Aghcheh and Kubicek, 2015). It forms a heterotrimeric with the VelB and VelA of the velvet family proteins (VelB-VelA-LaeA complex), that coordinate the light signal-dependent fungal development linked to secondary metabolism (Fischer et al., 2016; Chang et al., 2019). The presence of a methyltransferase domain in LaeA is associated with the regulation of secondary metabolite gene cluster expression through the epigenetic modification of the chromatin structure (Perrin et al., 2007). The LaeA protein is thought to facilitate the repression of the heterochromatin *via* association with either heterochromatin-associated methylases or deacetylases (Shwab and Keller, 2008; Kosalková et al., 2009).

LaeA in chromatin remodeling is a global regulator whose role is mainly facilitated by histone methylation (Wu and Yu, 2015). Deletion of *laeA* blocked the expression of sterigmatocystin, while its overexpression triggered an increased gene transcription of certain products such as penicillin and lovastatin in various *Aspergillus* spp. (Bok and Keller, 2004; Wu and Yu, 2015). Deletion of *hdaA*, a methyltransferase from *Aspergillus* that is a homologous of LaeA histone HDAC, increased secondary metabolite production (Okada and Seyedsayamdost, 2017). Further evidence indicated that the deletion of *hdaA*, in the fungal endophyte, *Calcarisporium arbuscular* led to the activation of ten compounds. Four of these were novel structures, including three tricyclic diterpenes and a novel meroterpenoid (Mao et al., 2015; Ding et al., 2020). LaeA protein is thus considered a global regulator of secondary metabolism whose levels modify the profile of secondary metabolites production (Lim et al., 2012).

Bok et al. (2009) hypothesized that in addition to LaeA protein, other chromatin-modifying proteins such as the members of the COMPASS (complex associated with Set1) also play a vital role in regulating the biosynthetic gene clusters. They identified *CclA* from *A. nidulans*, an ortholog of Bre2 in *Saccharomyces cerevisiae*. This gene was deleted together with a gene encoding a fatty acid synthase (stcJ) previously identified in *A. nidulans*, which is required for sterigmatocystin production (Bok and Keller, 2004; Bok et al., 2006, 2009). The double mutant strain could not produce sterigmatocytin. However, it was able to produce two known compounds (*A. nidulans* austinol and dehydroaustinol) and six aromatic compounds not previously identified in this fungus, and they were identified as monodictyphenone, emodin and four emodin analogs. All the compounds produced shared a similar aromatic non-reduced polyketide structure (Bok et al., 2009). The authors targeted ten unknown non-reduced polyketide clusters and were able to link polyketides responsible for producing all the identified compounds (Bok et al., 2009).

### Chromatin Remodeling Alters the Profile of Fungal Secondary Metabolites

Following this discovery that the disruption of histone deacetylase activity led to the transcriptional activation of gene clusters encoding for sterigmatocystin and penicillin, Shwab et al. (2007) showed that histone-modifying enzymes influence secondary metabolism. They found that HDAC mutants of *A. nidulans* generated by the deletion of *hdaA* bypassed the regulation by *laeA*. The genetic depletion of *hdaA* resulted in an increased expression of subtelomeric secondary metabolites, sterigmatocystin and penicillin (Shwab et al., 2007; Gacek and Strauss, 2012). These results provided strong evidence that *hdaA* mediate the expression of subtelomeric secondary metabolite gene clusters in *Aspergillus.* The role of *hdaA* as a global suppresser of biosynthetic genes was postulated to be a common occurrence since this HDAC is conserved in filamentous fungi (Shwab et al., 2007; Mao et al., 2015). Indeed, deletion of *hdaA* in filamentous fungi such as *Calcarisporium arbuscular* (Mao et al., 2015), *Penicillium chrysogenum* Fes1701 (Ding et al., 2020) resulted in a pleiotropic activation of secondary metabolites. Although this gene is an obvious target for activating secondary metabolite production, its deletion often results in other development defects such as slower growth, shorter mycelia, and defective sporulation (Mao et al., 2015). For example, in a study by Niu et al. (2015), they observed a decrease in conidia production upon the deletion of a putative histone deacetylase gene, *hid1* in *Pestalotiopsis microspore* NK17. The deletion of this HDAC gene resulted in a twofold increase pestalotiollide B yield. These observations are expected since HDACs are directly or indirectly involved in the development, proliferation, differentiation, and cell death (Kim and Bae, 2011; Niu et al., 2015).

The results observed after genetic depletion of HDAC suggested that pharmacological depletion using chemical HDAC inhibitors (known as an epigenetic modification) could provide means of regulating or increasing secondary metabolite production without genetic deletions (Shwab et al., 2007; Zutz et al., 2014). This strategy has since been popular in inducing a wide variety of novel secondary metabolites that are otherwise transiently expressed by fungi or have never been detected due to silencing of the encoding biosynthetic gene (Shwab et al., 2007). Interestingly, fungal species are primed for chromatin remodeling since their biosynthetic genes group together in locations that tend to be near the telomeres of their chromosomes (Toghueo et al., 2020). This localization of biosynthetic gene clusters near telomeres of the chromosomes might be a setup to facilitate efficiently coordinated regulation through chromatin remodeling within the subtelomeric regions (Brosch et al., 2008).

Epigenetic modification of the chromatin structure has shown to be a powerful tool in activating silent genes (Reyes-Dominguez et al., 2010; Strauss and Reyes-Dominguez, 2011; Chávez et al., 2015). In a vast number of cases, it has also led to the production of novel bioactive molecules. There are three levels involved in epigenetic regulation (i) DNA methylation, essential for normal development and differentiation. Transcription is inhibited by the covalent addition of a methyl group at the 5-C of the cytosine ring resulting in a 5-methylcytosine (5-mC) which extends into the major groove of DNA (Aghcheh and Kubicek, 2015); (ii) chromatin remodeling by histone modification where histones can undergo post-translational modifications (PTMs), which alter the interaction with DNA and nuclear proteins (Bannister and Kouzarides, 2011; Aghcheh and Kubicek, 2015); and (iii) RNA interference where non-coding RNA (ncRNA), which are non-translated into proteins, regulate transcriptional and post-transcriptional gene expression (Aghcheh and Kubicek, 2015). Two groups of ncRNA have been identified; short ncRNA (subcategorized into microRNA, short interfering RNA, and piwi-interacting RNA) and long ncRNA (Aghcheh and Kubicek, 2015). Short interfering RNA binds to specific target messenger RNA and inhibits translation by inducing its degradation, thus silencing post-transcriptional modification (Aghcheh and Kubicek, 2015; Collemare and Seidl, 2019).

This review will focus on epigenetic modification of the chromatin employing small molecule effectors. In a study by Nützmann et al. (2013), they demonstrated, using *Aspergillus nidulans*, that chemical inhibitors can lead to the upregulation of secondary metabolite gene clusters, with the possible alteration in the production of secondary metabolites of the targeted activation or inactivation of specific chromatin modifiers (König et al., 2013). The use of HDAC inhibitors like valproic acid and suberoylanilide hydroxamic acid (SAHA) led to the increased production of secondary metabolites in fungi. This inhibition of the HDACs has been attributed to the hyperacetylation of chromatin, which subsequently opens the chromatin (Beau et al., 2012; Takahashi et al., 2016). Several studies show the effects of treatment of fungi in culture media with these small molecule inhibitors and the changes in the expression profile of SM gene clusters (Cichewicz, 2010; Collemare and Seidl, 2019). The profile of metabolites detected in the broths (Williams et al., 2008; Chung et al., 2013) has allowed for the isolation of previously unknown compounds such as nygerone A from *Aspergillus niger* (Henrikson et al., 2009) and new sesquiterpenoids from *Aspergillus sydowii* (Chung et al., 2013). Since this strategy does not require genome sequencing, it may be suitable as a tool for initial metabolite screening of fungi from extreme environments, in combination with OSMAC strategies. However, there is no significant alteration of the metabolic profile observed after treatment in some cases, indicating that this strategy may not be applicable in all fungal species. For example, fungi such as *Alternaria brassicicola* and *Diheretospora chlamydosporia* were resistant to HDAC inhibitors (Brosch et al., 2008). The resistance may probably be due to that other filamentous fungus producing HDAC inhibitors allowing the fungus to escape inhibition.

## Mechanisms of Action of Small Molecular Compounds, Target Sites, Advantages, and Disadvantages

Epigenetic modifiers or epidrugs are natural or synthetic small molecular compounds targeting the epigenetic marks or enzymes with epigenetic activity leading to epigenetic alterations (Poças-Fonseca et al., 2020). Many of these compounds act by inhibiting enzyme machinery essential for transferring methyl, acetyl, or alkyl groups to DNA or histones (Table 1). In the past two decades, vast and diverse compounds have been produced by treating fungi with epigenetic modifiers (Williams et al., 2008; Linnakoski et al., 2018; Toghueo et al., 2020). Chromatin remodeling using epigenetic modifiers such as histone deacetylase (HDAC) and DNA methyltransferase (DNMT) inhibitors presents a straightforward and low-cost approach to uncovering the regulatory circuit in fungal biosynthetic genomes. The fungal biosynthetic genes hold a promise as a reservoir for novel chemistries that could be developed into pharmaceuticals (Gacek and Strauss, 2012).

**TABLE 1:** Small molecular compounds and their mechanism of action.

Mechanism of action	Epigenetic elicitor	Target site	References
Inhibition of HDAC class 1 and 2	Sodium butyrate	Heterochromatin	Vrba et al., 2011
	*Valproic acid*		Krämer et al., 2003
	SAHA		Henrikson et al., 2009
	Trichostatin A (TSA)		Smith and Edlind, 2002
Inhibition of DNA methyltransferase	5-Azacytidine	DNA	Williams et al., 2008; Wang et al., 2010
	Hydralazine hydrocloride		Cichewicz, 2010
Inhibition of proteosome	Bortezombid	Proteasomes	VanderMolen et al., 2014

Histone deacetylases inhibitors are structurally classified into four classes: hydroxamates, cyclic peptides, aliphatic acids, and benzamides b (reviewed by Kim and Bae, 2011). HDAC inhibitors alter gene expression patterns and endorse changes in non-histone proteins occurring at the post-translational level (Shwab et al., 2007; Williams et al., 2008). Trichostatin A (TSA), its derivative, suberoylanilide hydroxamic acid (SAHA), and sodium butyrate are frequently used as HDAC inhibitors in filamentous fungi. Both TSA and SAHA present a hydroxamic group that binds to the zinc ion of class 1 and 2 classical HDAC’s active sites, thus preventing their activities. Sodium butyrate inhibits the histone deacetylase activity, leading to differentiation in eukaryotic cells (Zutz et al., 2014; Poças-Fonseca et al., 2020). Class 1 and class 2 classical HDAC inhibitor, TSA, and other HDAC chemical inhibitors such as SAHA, sodium butyrate, and valproic acid have been shown to enhance the chemical diversity of secondary metabolites produced by fungi from the genera *Clonostachys, Diatrype*, and *Verticillium* (Williams et al., 2008). Valproic acid is also frequently used, and it inhibits class 1 classical HDACs and also induces proteosomal degradation of class2 classical HDACs (Zutz et al., 2014, 2016).

DNA methyltransferases (DNMTs) are a conserved family of cytosine methylases that play an essential role in maintaining DNA methylation patterns, transcriptional activation, and silencing (Lyko, 2018). DNMT inhibitors: 5-azacytidine (5-AZA) and decitabine (5-AZA-2′-deoxycytidine) are synthetic analogs of cytidine presenting nitrogen instead of carbon in the “5” of the pyrimidine ring. This ligand is incorporated in the DNA and less into RNA, preventing the proper transferring of the methyl group by the DNMT (Zutz et al., 2014). Inhibition by DNMT inhibitors results in passive demethylation through consecutive DNA replication cycles. In the presence of DNMT, DNMTs remain bound to the DNA and are degraded by the proteasome pathway (Poças-Fonseca et al., 2020).

In Table 2, we present a comprehensive summary of the application of HDAC and DNMT inhibitors in various fungal species. We show that a variety of chemical diversity, instead of silenced in untreated fungal species, can be produced upon treatment with different pan inhibitors. The use of pan inhibitors for inducing BGC expression is a vital tool for screening fungal environmental isolates without any prior knowledge of their genetic information. However, treatment with small molecular compounds often lead to a pleiotropic activation, making it difficult to predict which secondary metabolites will be produced and to link the producing gene to a particular phenotype. Although the approach of using small molecular epigenetic modifiers yields diverse chemistries, many studies do not assess the activity profiles of these compounds. This challenge can be attributed to limited access to standardized *in vitro* methods for activity testing in different fungal natural products laboratories. This highlights a need for collaborations and the adoption of a standard workflow to discover secondary metabolites from natural resources such as fungi.

**TABLE 2:** Epigenetic induction of fungal biosynthetic gene clusters encoding secondary metabolites using small molecular compounds.

Fungal species	Culture conditions	Epigenetic modifier	Secondary metabolites produced	Activity profile	References
*Cladosporium cladosporioides*	Potato dextrose broth, 25°C, shaking at 100 rpm for 24 h, added elicitor and further incubation for 6 days	5-azacytidine, concentration range (0.1 μM-10 mM). used 10-fold less than MIC	three oxylipins [(9Z,12Z)-11-hydroxyoctadeca-9,12-dienoic acid, its methyl ester, and glycerol conjugate]	N/A	Williams et al., 2008
		SAHA, concentration range (0.1 μM-10 mM). used 10-fold less than MIC	perylenequinones, four known cladochromes (A, B, D and E) and two novel cladochromes (F and G), calphostin B	N/A	
*Diatrype disciformis*		5-azacytidine, concentration range (0.1 μM-10 mM). used 10-fold less than MIC	two new polyketides (lunalides A and B)	N/A	
*Aspergillus niger ATCC 1015*	Semi-solid vermiculite-based media#, 25°C, static condition (12 h light and 12 h dark) for 2 weeks	10 μM SAHA	nygerone A	N/A	Henrikson et al., 2009
*Penicillium citreonigrum*	Semi-solid vermiculite-based media#, 20°C, static conditions for 20 days	50 μM 5-azacytidine	six azaphilones (sclerotiorin, sclerotioramine, ochrephilone, dechloroisochromophilone 111, dechloroisochromophilone IV, 6-[(3E,5E)-5,7-dimethyl-2-methlenenona-3,5-dienyl]-2,4-dihydroxy-3-methylbenzaldehyde, pencolide) and two new meroterpenes (atlantinones A and B)	-moderate inhibition of *Staphylococcus epidermidis* (sclerotionin and sclerotioramine) -inhibition of Candida strains (sclerotioramine)	Wang et al., 2010
*Leucostoma persoonia*	Sabouraud dextrose agar, 30°C, static conditions for 3 weeks	100 μM sodium butyrate	enhanced production of cytosporones (B, C, and E) and unknown cytosporone R	cytosporone E active against *Plasmodium falciparum* and MRSA with low cytotoxicity in mammalian cells	Beau et al., 2012
		50 μM 5-azacytidine	• novel compound cytosporone R • enhanced production of known cytosporones B (360%), C (580%), and E (890%)		
*Alternaria sp.*	Potato dextrose broth, 30°C, static conditions for 7 days	250 μM 5-azacytidine	mycotoxins including alternariol, alternariol-5-O-methyl ether, 3′-hydroxyalternariol-5-O-methyl ether, altenusin, tenuazonic acid, and altertoxin II.	N/A	Sun et al., 2012
		500 μM SBHA			
*Aspergillus clavatus*	Fungal minimum media 1 (FM1): tryptic digested casein peptone or Fungal minimum media 2 (FM2): papaine-degested peptide	5 μM valproic acid	increased production of cytochalasin E (FM2 media), increased production of Pseurotin A (FM1 media) at 48 and 72 h	N/A	Zutz et al., 2013
		5 μM trichostin A	increased production of cytochalasin E (FM2 media), increase in Pseurotin A (FM1 media) at 48 h		
		5 μM sodium butyrate			
		5 μM 5-azacytidine	increased production of cytochalasin E (FM2 media), increase in Pseurotin A (FM1 media) at 72 h		
*Chaetomium indicum*	Potato dextrose broth, 25°C, shaking at 150 rpm for 16 days	500 μM SAHA	enhanced the production of six novel prenylated aromatic polyketides, chaetophenols (A–F)	N/A	Asai et al., 2013
*Chaetomium cancroideum*	Potato dextrose broth, 25°C, shaking at 150 rpm for 14 days	50 μM nicotinamide	Three polyketides (chaetophenol G, cancrolides A and B)	N/A	Asai et al., 2016
*Pestalotiopsis acacia*	Potato dextrose broth, 30°C, shaking conditions for 7 days	500 μM SBHA and 500 μM 5-azacitidine	three novel aromatic compounds 1. 20-hydroxy-60-hydroxymethyl-40-methylphenyl-2,6-dihydroxy-3-(2-isopentenyl)benzoate; 2. 4,6-dihydroxy-7-hydroxymethyl-3-methylcoumarin 3. 4,6-dihydroxy-3,7-dimethylcoumarin five known polyketides (endocrocin, pestalotiollide B, pestalotiopyrone G, scirpyrone A and 7-hydroxy-2-(2-hydroxypropyl)-5-methylchromone.	N/A	Yang et al., 2013
*Endophytic Fusarium oxysporum strain R1*	Potato dextrose broth, 28°C, static conditions for 7 days	500 μM SBHA	novel fusaric acid derivative: 5-Butyl-6-oxo-1,6-dihydropyridine-2-carboxylic acid and 5-(But-9-enyl)-6-oxo-1,6-dihydropyridine-2-carboxylic acid	both fusaric acid derivatives showed no activity against *Bacillus cereus* while control (fusaric) shown activity	Chen et al., 2013
*Penicillium funiculosum*	Potato dextrose broth, 25°C, shaking conditions at for 2 weeks	100 μM 5-Azacytidine	induced the biosynthesis of two new prenyleudesmane diterpeniods	-no toxicity against three human cancer cell lines -not antimicrobial activity against *Staphylococcus aureus*, *Bacillus thuringiensis*, and *B. subtilis*	Liu et al., 2014
*Chaetomium* sp.	Milk Rice (Milch-Reis, ORYZA), 23°C, static conditions for 3–4 weeks. 2-days pre-incubation before adding elicitors	6 mM 5-azacytidine	induction of two new peaks compared to control, one identified as isosulochrin	N/A	Akone et al., 2016
		6 mM SAHA			
*Penicillium brevicompactum*	Malt extract broth, 30°C, static conditions for 4 weeks	100 μM nicotinamide	p-anisic acid, p-anisic acid methyl ester, benzyl anisate, syringic acid, sinapic acid, acetosyringone, phenyl acetic acid, gentisaldehyde, and p-hydroxy benzaldehyde	N/A	El-Hawary et al., 2018
		0.01 M Sodium butyrate	enhanced the production of anthranilic acid and ergosterol peroxide	N/A	
*Cochliobolus lunatus (TA26-46)*	Czapek Dox medium, Room temperature, 30 days	10 μM 5-Azacytidine	two new pyrones, (cochliobopyrones A and B), along with three isocoumarins and one chromone.	N/A	Wu et al., 2019
*Penicillium brasilianum*	Potato dextrose broth, 30°C, shaking at 70 rpm for 7 days	200 μM SAHA	Reduction of metabolites production such as brasiliamide A	N/A	Akiyama et al., 2020
		Nicotinamide			
*Aspergillus* *Calidoustus*	Aspegillus complete medium (2.5% glucose, 0.5% yeast extract), 25°C, shaking at 150 rpm for 7 days	100 μM Vorinostat (or SAHA)	emericellamide A, emericellamide B, increased titer of phenylahistin	N/A	Aldholmi et al., 2020

*SAHA, suberanilohydroxamic acid; SBHA, suberoylanilide hydroxamic acid; HDAC, histone deacetylase; DNMT, DNA methyltransferase; N/A, not available.*

## Response to Epigenetic Treatment is Complex at a Metabolome Level

Histone post-translational modifications (PTMs) such as acetylation and methylation play a profound role in chromatin-based regulation of secondary metabolite (SM) gene clusters (Gacek-Matthews et al., 2016). However, the exact molecular mechanism mediated by histone PTMs leading to the activation or repression of SM gene clusters is not yet fully understood. Studies in the model organism, *Aspergillus nidulans*, revealed that SM gene clusters are under chromatin-based regulatory control (Shwab et al., 2007). Subsequent studies in HDAC inhibition and SAGA-complex mutants showed the importance of acetylation in the expression of these SM gene clusters (Gacek and Strauss, 2012). Consequently, genetic deletion of, HDACs in various fungal species such as *Aspergillus fumigatus, Aspergillus oryzae, Fusarium fujikuroi, Penicillium oryzae, Fusarium asiaticum, P. chrysogenum*, and *A. nidulans* have resulted in increased expression of known gene clusters and expression of novel clusters that are repressed in the control strain (Albright et al., 2015; Collemare and Seidl, 2019).

Generally, acetylation of histones (e.g., H3K27ac in *Neurospora crassa*) is associated with transcriptional activation of SM gene clusters (Ferraro et al., 2021; Zhang et al., 2021). Although the exact mechanism of this activation is not known, there are various proposed mechanisms suggested by observations of histone marks in different genetic analysis studies (Gacek-Matthews et al., 2016; Ferraro et al., 2021). The earlier proposed mechanism is that histone marks such as acetylation of histones neutralizes the positive charge of the ε-amino group of lysine in the nucleosome, thus weakening the interaction between the core histones with negatively charged DNA and subsequent chromatic accessibility (Gacek-Matthews et al., 2016). Another possibility is that the acetylation of lysines alters the interaction of regulatory proteins with histones (Brosch et al., 2008) or recruit chromatin-modifying enzymes that promote or inhibit transcription of chromatin-regulated gene clusters (Gacek-Matthews et al., 2016).

In addition to the specific modification site, the complex combination of distinct histone modifications creates a pattern that is likely recognized and interpreted as a modification signal by regulatory factors (Brosch et al., 2008; Pidroni et al., 2018). For example, increased SM production has been associated with the global increase of H3K14ac and SM gene cluster-specific H3K9ac (Nützmann et al., 2013; Collemare and Seidl, 2019). However, in a study by Gacek-Matthews et al. (2016), they reported that histone marks associated with activation were observed in some transcribed clusters of *A. nidulans*, but this was not true for most of the transcribed gene clusters. These observations suggest that chromatin-based regulation is more complex and depends on many interacting factors. Indeed, the histone modifications do not occur independently but are interrelated, thus influencing the biological outcome (Brosch et al., 2008). In a study by Rea et al. (2000), the effect of functional interdependence was clear where the phosphorylation of Serine 10 in H3 was linked to facilitating the acetylation of H3k9 and H3K4. They also observed that H3K9 methylation inhibited phosphorylation, thus suggesting that chromatin networks are rather regulated by dynamic mechanisms challenging to elucidate *in vitro* (Brosch et al., 2008).

The complex regulatory nature of the chromatin has been observed also in genetic depletion studies probing the role of HDACs in secondary metabolite production. Evidence from these studies suggest that while the deletion of HDACs may lead to the activation of previously silenced biosynthetic gene clusters, a subset of genes may also be repressed by the genetic deletion of HDACs (Brosch et al., 2008; Pfannenstiel and Keller, 2019). Evidence of this differential response was first shown by Albright et al. (2015), where they used untargeted metabolomics to profile >1000 small molecules following genetic or chemical reductions in histone deacetylase activity. They found that *A. nidulans* could increase its capacity to produce more than 61 compounds, including a lipopeptide aldehyde, fellutamides known as a proteasome inhibitor by ∼100-fold upon inhibition of a single HDAC, *RpdA*. Interestingly, there was a simultaneous decrease in the expression of 47 compounds by >100-fold. Further evidence of differential expression has been recently reported by Ding et al. (2020), where deletion of *HdaA* from an endophytic fungus, *Penicillium chrysogenum* FES1701. These researchers observed that in the *HdaA* mutant strain, the BGC encoding the meleagrin/roquefortine was upregulated while the chrysogine encoding BGC was downregulated. Interestingly, a similar pattern has been observed upon treatment of fungal species with HDAC inhibitors. Magotra et al. (2017) reported a 10-fold upregulation of fumiquinazoline C when *Aspergillus fumigatus* (GA-L7), an endophyte of *Grewia asiatica* L. was treated with valproic acid, a class I HDAC inhibitor that also induces the proteosomal degradation of class II HDACs (Krämer et al., 2003). In the same study, a reduction was observed in the production of seven other metabolites produced under normal conditions. These observations provide evidence that the overall response to HDAC inhibition at the level of secondary metabolome is more complex than the currently assumed global increase in the abundance of secondary metabolites (Albright et al., 2015).

## Future Perspectives

This review highlights the influence of epigenetic modifiers on rearranging the chromatin network, thus ensuring fungal ability to produce secondary metabolites. Epigenetic modification using small molecular modulators offers a convenient tool for the initial screening of novel fungal isolates such as those from the less explored environments. The discovery that endophytic fungi can produce similar metabolites to their host plants places the endophytes as attractive candidates for future drug discoveries. Moreover, bioprospecting plant endophytes instead of plants provide a sustainable alternative as plants are prone to extinction due to overharvesting. Over recent years there has been a remarkably rapid development of new techniques for biosynthetic gene clusters used in drug discovery. Low molecular weight molecules such as 5-AZA, SAHA, and valproic acid can induce secondary metabolite production in fungi by displaying chromatin remodeling. This method provides a considerable potential for inducing a vast number of bioactive compounds. It has been found that each low molecular weight molecule can induce different patterns of secondary metabolite production in the same fungal culture species. These findings can assist in optimizing secondary metabolite production using epigenetic modifiers as an economical, non-invasive approach. However, future studies need to establish the exact mechanism by which these small molecular inhibitors facilitate the changes in the chromatin network. Establishing the mechanism of action will enable a more targeted approach that results in a specific expression of compounds of interest.

## Author Contributions

SIN conceptualized, critically reviewed, and added value to the overall manuscript. LCP and SIN wrote the manuscript, gathered literature, and made necessary edits after critical reviews. LN and PJM were involved in the literature collection and contributed to writing the section on small molecular modifiers. All authors contributed to the article and approved the submitted version.

## Conflict of Interest

The authors declare that the research was conducted in the absence of any commercial or financial relationship that could be construed as a potential conflict of interest.

## Publisher’s Note

All claims expressed in this article are solely those of the authors and do not necessarily represent those of their affiliated organizations, or those of the publisher, the editors and the reviewers. Any product that may be evaluated in this article, or claim that may be made by its manufacturer, is not guaranteed or endorsed by the publisher.

## References

[B1] Aghcheh R. K. Kubicek C. P. (2015). Epigenetics as an emerging tool for improvement of fungal strains used in biotechnology. *Appl. Microbiol. Biotechnol*. 99 6167–6181. 10.1007/s00253-015-6763-2 26115753

[B2] Akiyama D. Y. Rocha M. C. Costa J. H. Malavazi I. Fill T. P. (2020). The histone deacetylase clr3 regulates secondary metabolite production and growth under oxidative stress conditions in *Penicillium brasilianum*. *bioRxiv* [Preprint]. 10.1101/2020.05.01.072108 PMC9146837 35628769

[B3] Akone S. H. Mandi A. Kurtan T. Hartmann R. Lin W. Daletos G. (2016). Inducing secondary metabolite production by the endophytic fungus *Chaetomium* sp. through fungal-bacterial co-culture and epigenetic modification. *Tetrahedron* 72 6340–6347.

[B4] Albright J. C. Henke M. T. Soukup A. A. McClure R. A. Thomson R. J. Keller N. P. (2015). Large-scale metabolomics reveals a complex response of *Aspergillus nidulans* to epigenetic perturbation. *ACS Chem. Biol.* 10 1535–1541. 10.1021/acschembio.5b00025 25815712 PMC4475433

[B5] Aldholmi M. Wilkinson B. Ganesan A. (2020). Epigenetic modulation of secondary metabolite profiles in *Aspergillus calidoustus* and *Aspergillus westerdijkiae* through histone deacetylase (HDAC) inhibition by vorinostat. *J. Antibiot.* 73 410–413. 10.1038/s41429-020-0286-5 32060485

[B6] Asai T. Morita S. Taniguchi T. Monde K. Oshima Y. (2016). Epigenetic stimulation of polyketide production in *Chaetomium cancroideum* by an NAD(+)-dependent HDAC inhibitor. *Org. Biomol. Chem*. 14 646–651. 10.1039/c5ob01595b 26549741

[B7] Asai T. Yamamoto T. Shirata N. Taniguchi T. Monde K. Fujii I. (2013). Structurally diverse chaetophenol productions induced by chemically mediated epigenetic manipulation of fungal gene expression. *Org. Lett*. 15 3346–3349. 10.1021/ol401386w 23767797

[B8] Bannister A. J. Kouzarides T. (2011). Regulation of chromatin by histone modifications. *Cell Res*. 21 381–395. 10.1038/cr.2011.22 21321607 PMC3193420

[B9] Baral B. Akhgari A. Metsä-Ketelä M. (2018). Activation of microbial secondary metabolic pathways: avenues and challenges. *Synth. Syst. Biotechnol*. 3 163–178. 10.1016/j.synbio.2018.09.001 30345402 PMC6190515

[B10] Beau J. Mahid N. Burda W. N. Harrington L. Shaw L. N. Mutka T. (2012). Epigenetic tailoring for the production of anti-infective cytosporones from the marine fungus *Leucostoma persoonii*. *Mar. Drugs* 10 762–774. 10.3390/md10040762 22690142 PMC3366674

[B11] Begani J. Lakhani J. Harwani D. (2018). Current strategies to induce secondary metabolites from microbial biosynthetic cryptic gene clusters. *Ann. Microbiol.* 68 419–432. 10.1007/s13213-018-1351-1

[B12] Berg G. Hallmann J. (2006). “Control of Plant Pathogenic Fungi with Bacterial Endophytes,” in *Microbial Root Endophytes. Soil Biology, vol 9*, eds Schulz B. J. E. Boyle C. J. C. Sieber T. N. (Berlin: Springer), 10.1007/3-540-33526-9_4

[B13] Bertrand S. Bohni N. Schnee S. Schumpp O. Gindro K. Wolfender J. L. (2014). Metabolite induction via microorganism co-culture: a potential way to enhance chemical diversity for drug discovery. *Biotechnol. Adv*. 32 1180–1204. 10.1016/j.biotechadv.2014.03.001 24651031

[B14] Bode H. B. Bethe B. Höfs R. Zeeck A. (2002). Big effects from small changes: possible ways to explore nature’s chemical diversity. *Chem. Biochem.* 3 619–627. 10.1002/1439-7633(20020703)3:7<619::AID-CBIC619<3.0.CO;2-9 12324995

[B15] Bok J. W. Chiang Y. M. Szewczyk E. Reyes-Dominguez Y. Davidson A. D. Sanchez J. F. (2009). Chromatin-level regulation of biosynthetic gene clusters. *Nat. Chem. Biol.* 5 462–464. 10.1038/nchembio.177 19448638 PMC2891026

[B16] Bok J. W. Keller N. P. (2004). LaeA, a regulator of secondary metabolism in *Aspergillus spp*. *Eukaryot. Cell* 3 527–535. 10.1128/EC.3.2.527-535.2004 15075281 PMC387652

[B17] Bok J. W. Noordermeer D. Kale S. P. Keller N. P. (2006). Secondary metabolic gene cluster silencing in *Aspergillus nidulans*. *Mol. Microbiol.* 61 1636–1645. 10.1111/j.1365-2958.2006.05330.x 16968230

[B18] Brosch G. Loidl P. Graessle S. (2008). Histone modifications and chromatin dynamics: a focus on filamentous fungi. *FEMS Microbiol. Rev.* 32 409–439. 10.1111/j.1574-6976.2007.00100.x 18221488 PMC2442719

[B19] Calvo A. M. Wilson R. A. Bok J. W. Keller N. P. (2002). Relationship between secondary metabolism and fungal development. *Microbiol. Mol Biol. Rev*. 66 447–459. 10.1128/MMBR.66.3.447-459.2002 12208999 PMC120793

[B20] Card S. Johnson L. Teasdale S. Caradus J. (2016). Deciphering endophyte behaviour: the link between endophyte biology and efficacious biological control agents. *FEMS Microbiol. Ecol.* 92:fiw114. 10.1093/femsec/fiw114 27222223

[B21] Caterino T. L. Hayes J. J. (2011). Structure of the H1 C-terminal domain and function in chromatin condensation. *Biochem. Cell Biol.* 89 35–44. 10.1139/O10-024 21326361 PMC3787537

[B22] Chang Z. Yadav V. Lee S. C. Heitman J. (2019). Epigenetic mechanisms of drug resistance in fungi. *Fungal Genet. Biol.* 132:103253. 10.1016/j.fgb.2019.103253 31325489 PMC6858951

[B23] Chávez R. Fierro F. García-Rico R. O. Vaca I. (2015). Filamentous fungi from extreme environments as a promising source of novel bioactive secondary metabolites. *Front. Microbiol.* 6:903. 10.3389/fmicb.2015.00903 26441853 PMC4563253

[B24] Chen H. J. Awakawa T. Sun J. Y. Wakimoto T. Abe I. (2013). Epigenetic modifier-induced biosynthesis of novel fusaric acid derivatives in endophytic fungi from *Datura stramonium* L. *Nat. Prod. Bioprospect.* 3 20–23. 10.1007/s13659-013-0010-2

[B25] Chiang Y. M. Lee K. H. Sanchez J. F. Keller N. P. Wang C. C. (2009). Unlocking fungal cryptic natural products. *Nat. Prod. Commun.* 4 1505–1510. 19967983 PMC3101174

[B26] Chung Y. M. Wei C. K. Chuang D. W. El-Shazly M. Hsieh C. T. Asai T. (2013). An epigenetic modifier enhances the production of anti-diabetic and anti-inflammatory sesquiterpenoids from *Aspergillus sydowii*. *Bioorg. Med. Chem.* 21 3866–3872. 10.1016/j.bmc.2013.04.004 23647825

[B27] Cichewicz R. H. (2010). Epigenome manipulation as a pathway to new natural product scaffolds and their congeners. *Nat. Prod. Rep.* 27 11–22. 10.1039/b920860g 20024091 PMC2958777

[B28] Collemare J. Seidl M. F. (2019). Chromatin-dependent regulation of secondary metabolite biosynthesis in fungi: is the picture complete?. *FEMS Microbiol. Rev.* 43 591–607. 10.1093/femsre/fuz018 31301226 PMC8038932

[B29] Deepika V. B. Murali T. S. Satyamoorthy K. (2016). Modulation of genetic clusters for synthesis of bioactive molecules in fungal endophytes: a review. *Microbiol. Res.* 182 125–140. 10.1016/j.micres.2015.10.009 26686621

[B30] Ding Z. Zhou H. Wang X. Huang H. Wang H. Zhang R. (2020). Deletion of the Histone Deacetylase HdaA in Endophytic Fungus *Penicillium chrysogenum* Fes1701 Induces the Complex Response of Multiple Bioactive Secondary Metabolite Production and Relevant Gene Cluster Expression. *Molecules* 25:3657. 10.3390/molecules25163657 32796640 PMC7464707

[B31] El-Hawary S. S. Sayed A. M. Mohammed R. Hassan H. M. Zaki M. A. Rateb M. E. (2018). Epigenetic Modifiers Induce Bioactive Phenolic Metabolites in the Marine-Derived Fungus *Penicillium brevicompactum*. *Mar. Drugs* 16:253. 10.3390/md16080253 30061488 PMC6117726

[B32] Fadiji A. E. Babalola O. O. (2020). Elucidating Mechanisms of Endophytes Used in Plant Protection and Other Bioactivities with Multifunctional Prospects. *Front. Bioeng. Biotechnol.* 8:467. 10.3389/fbioe.2020.00467 32500068 PMC7242734

[B33] Fan A. Mi W. Liu Z. Zeng G. Zhang P. Hu Y. (2017). Deletion of a Histone Acetyltransferase Leads to the Pleiotropic Activation of Natural Products in *Metarhizium robertsii*. *Org. Lett.* 19 1686–1689. 10.1021/acs.orglett.7b00476 28301168

[B34] Ferraro A. R. Ameri A. J. Lu Z. Kamei M. Schmitz R. J. Lewis Z. A. (2021). Chromatin accessibility profiling in *Neurospora crassa* reveals molecular features associated with accessible and inaccessible chromatin. *BMC Genomics* 22:459. 10.1186/s12864-021-07774-0 34147068 PMC8214302

[B35] Fischer J. Schroeckh V. Brakhage A. A. (2016). “Awakening of fungal secondary metabolite gene clusters,” in *Gene Expression Systems in Fungi: Advancements and Applications*, eds Schmoll M. Dattenböck C. (Cham: Springer), 253–273.

[B36] Fox E. M. Howlett B. J. (2008). Secondary metabolism: regulation and role in fungi biology. *Curr. Opin. Microbiol.* 11 481–487. 18973828 10.1016/j.mib.2008.10.007

[B37] Fyodorov D. V. Zhou B. R. Skoultchi A. I. Bai Y. (2018). Emerging roles of linker histones in regulating chromatin structure and function. *Nat. Rev. Mol. Cell Biol.* 19 192–206. 10.1038/nrm.2017.94 29018282 PMC5897046

[B38] Gacek A. Strauss J. (2012). The chromatin code of fungal secondary metabolite gene clusters. *Appl. Microbiol. Biotechnol.* 95 1389–1404. 10.1007/s00253-012-4208-8 22814413 PMC3427479

[B39] Gacek-Matthews A. Berger H. Sasaki T. Wittstein K. Gruber C. Lewis Z. A. (2016). KdmB, a Jumonji Histone H3 Demethylase, Regulates Genome-Wide H3K4 Trimethylation and Is Required for Normal Induction of Secondary Metabolism in *Aspergillus nidulans*. *PLoS Genet.* 12:e1006222. 10.1371/journal.pgen.1006222 27548260 PMC4993369

[B40] Giaimo B. D. Ferrante F. Herchenröther A. Hake S. B. Borggrefe T. (2019). The histone variant H2A.Z in gene regulation. *Epigenetics Chromatin* 12:37. 10.1186/s13072-019-0274-9 31200754 PMC6570943

[B41] González-Menéndez V. Crespo G. Toro C. Martín J. de Pedro N. Tormo J. R. (2019). Extending the Metabolite Diversity of the Endophyte *Dimorphosporicola tragani*. *Metabolites* 9:197. 10.3390/metabo9100197 31546616 PMC6835440

[B42] Guzmán-Chávez F. Zwahlen R. D. Bovenberg R. Driessen A. (2018). Engineering of the Filamentous Fungus *Penicillium chrysogenum* as Cell Factory for Natural Products. *Front. Microbial.* 9:2768. 10.3389/fmicb.2018.02768 30524395 PMC6262359

[B43] Hardoim P. R. van Overbeek L. S. Berg G. Pirttilä A. M. Compant S. Campisano A. (2015). The Hidden World within Plants: ecological and Evolutionary Considerations for Defining Functioning of Microbial Endophytes. *Microbiol. Mol. Biol. Rev*. 79 293–320. 10.1128/MMBR.00050-14 26136581 PMC4488371

[B44] Harvey C. J. B. Tang M. Schlecht U. Horecka J. Fischer C. R. Lin H. C. (2018). HEx: a heterologous expression platform for the discovery of fungal natural products. *Sci. Adv.* 4:5459. 10.1126/sciadv.aar5459 29651464 PMC5895447

[B45] Henrikson J. C. Hoover A. R. Joyner P. M. Cichewicz R. H. (2009). A chemical epigenetics approach for engineering the in situ biosynthesis of a cryptic natural product from *Aspergillus niger*. *Org Biomol. Chem.* 7 435–438. 10.1039/b819208a 19156306

[B46] Hewage R. T. Aree T. Mahidol C. Ruchirawat S. Kittakoop P. (2014). One strain-many compounds (OSMAC) method for production of polyketides, azaphilones, and an isochromanone using the endophytic fungus *Dothideomycete* sp. *Phytochemistry* 108 87–94. 10.1016/j.phytochem.2014.09.013 25310919

[B47] Jackson N. Czaplewski L. Piddock L. J. V. (2018). Discovery and development of new antibacterial drugs: learning from experience?. *J. Antimicrob. Chemother*. 73 1452–1459. 10.1093/jac/dky019 29438542

[B48] Jia M. Chen L. Xin H. L. Zheng C. J. Rahman K. Han T. (2016). A Friendly Relationship between Endophytic Fungi and Medicinal Plants: a Systematic Review. *Front. Microbiol.* 7:906. 10.3389/fmicb.2016.00906 27375610 PMC4899461

[B49] Jiang C. Lv G. Tu Y. Cheng X. Duan Y. Zeng B. (2021). Applications of CRISPR/Cas9 in the Synthesis of Secondary Metabolites in Filamentous Fungi. *Front. Microbiol.* 12:638096. 10.3389/fmicb.2021.638096 33643273 PMC7905030

[B50] Keller N. P. (2019). Fungal secondary metabolism: regulation, function and drug discovery. *Nat. Rev. Microbiol.* 17 167–180. 10.1038/s41579-018-0121-1 30531948 PMC6381595

[B51] Khare E. Mishra J. Arora N. K. (2018). Multifaceted Interactions Between Endophytes and Plant: developments and Prospects. *Front. Microbiol.* 9:2732. 10.3389/fmicb.2018.02732 30498482 PMC6249440

[B52] Kim H. J. Bae S. C. (2011). Histone deacetylase inhibitors: molecular mechanisms of action and clinical trials as anti-cancer drugs. *Am. J. Transl. Res.* 3 166–179. 21416059 PMC3056563

[B53] Kloepper J. W. Ryu C. M. (2006). “Bacterial Endophytes as Elicitors of Induced Systemic Resistance,” in *Microbial Root Endophytes*, eds Schulz B. J. E. Boyle C. J. C. Sieber T. N. (Berlin: Springer), 33–52. 10.1007/3-540-33526-9_3

[B54] König C. C. Scherlach K. Schroeckh V. Horn F. Nietzsche S. Brakhage A. A. (2013). Bacterium induces cryptic meroterpenoid pathway in the pathogenic fungus *Aspergillus fumigatus*. *Chem. Biochem.* 14 938–942. 10.1002/cbic.201300070 23649940

[B55] Kosalková K. García-Estrada C. Ullán R. V. Godio R. P. Feltrer R. Teijeira F. (2009). The global regulator LaeA controls penicillin biosynthesis, pigmentation and sporulation, but not roquefortine C synthesis in *Penicillium chrysogenum*. *Biochemie* 91 214–225. 10.1016/j.biochi.2008.09.004 18952140

[B56] Krämer O. H. Zhu P. Ostendorff H. P. Golebiewski M. Tiefenbach J. Peters M. A. (2003). The histone deacetylase inhibitor valproic acid selectively induces proteasomal degradation of HDAC2. *EMBO J.* 22 3411–3420. 10.1093/emboj/cdg315 12840003 PMC165640

[B57] Lamoth F. Juvvadi P. R. Steinbach W. J. (2015). Histone deacetylase inhibition as an alternative strategy against invasive aspergillosis. *Front. Mcrobiol.* 6:96. 10.3389/fmicb.2015.00096 25762988 PMC4329796

[B58] Lim F. Y. Sanchez J. F. Wang C. C. Keller N. P. (2012). Toward awakening cryptic secondary metabolite gene clusters in filamentous fungi. *Methods Enzymol.* 517 303–324. 10.1016/B978-0-12-404634-4.00015-2 23084945 PMC3703436

[B59] Linnakoski R. Reshamwala D. Veteli P. Cortina-Escribano M. Vanhanen H. Marjomäki V. (2018). Antiviral Agents From Fungi: diversity, Mechanisms and Potential Applications. *Front. Microbiol.* 9:2325. 10.3389/fmicb.2018.02325 30333807 PMC6176074

[B60] Liu D. Z. Liang B. W. Li X. F. Liu Q. (2014). Induced production of new diterpenoids in the fungus *Penicillium funiculosum*. *Nat. Prod. Commun.* 9 607–608. 25026698

[B61] Alvin A. Miller K. I Neilan B. A. (2014). Exploring the potential of endophytes from medicinal plants as sources of antimicrobial compounds. *Microbiol. Res.* 169 483–495. 24582778 10.1016/j.micres.2013.12.009 PMC7126926

[B62] Lyko F. (2018). The DNA methyltransferase family: a versatile toolkit for epigenetic regulation. *Nat. Rev. Genet.* 19 81–92. 10.1038/nrg.2017.80 29033456

[B63] Macheleidt J. Mattern D. J. Fischer J. Netzker T. Weber J. Schroeckh V. (2016). Regulation and Role of Fungal Secondary Metabolites. *Annu. Rev. Genet.* 50 371–392. 10.1146/annurev-genet-120215-035203 27732794

[B64] Magotra A. Kumar M. Kushwaha M. Awasthi P. Raina C. Gupta A. P. (2017). Epigenetic modifier induced enhancement of fumiquinazoline C production in *Aspergillus fumigatus* (GA-L7): an endophytic fungus from *Grewia asiatica* L. *AMB Express* 7:43. 10.1186/s13568-017-0343-z 28213885 PMC5315648

[B65] Mao X. M. Xu W. Li D. Yin W. B. Chooi Y. H. Li Y. Q. (2015). Epigenetic genome mining of an endophytic fungus leads to the pleiotropic biosynthesis of natural products. *Angew. Chem. Int. Ed. Engl.* 54 7592–7596. 10.1002/anie.201502452 26013262 PMC4487767

[B66] Martire S. Banaszynski L. A. (2020). The roles of histone variants in fine-tuning chromatin organization and function. *Nat. Rev. Mol. Cell Biol*. 21 522–541. 10.1038/s41580-020-0262-8 32665685 PMC8245300

[B67] Nair D. N. Padmavathy S. (2014). Impact of endophytic microorganisms on plants, environment and humans. *Sci. World J.* 2014:250693. 10.1155/2014/250693 24587715 PMC3920680

[B68] Netzker T. Fischer J. Weber J. Mattern D. J. König C. C. Valiante V. (2015). Microbial communication leading to the activation of silent fungal secondary metabolite gene clusters. *Front. Microbiol*. 6:299. 10.3389/fmicb.2015.00299 25941517 PMC4403501

[B69] Nielsen K. F. Larsen T. O. (2015). The importance of mass spectrometric dereplication in fungal secondary metabolite analysis. *Front. Microbiol.* 6:71. 10.3389/fmicb.2015.00071 25741325 PMC4330896

[B70] Niu X. Hao X. Hong Z. Chen L. Yu X. Zhu X. (2015). A Putative Histone Deacetylase Modulates the Biosynthesis of Pestalotiollide B and Conidiation in *Pestalotiopsis microspora*. *J. Microbiol. Biotechnol.* 25 579–588. 10.4014/jmb.1409.09067 25394605

[B71] Nützmann H. W. Fischer J. Scherlach K. Hertweck C. Brakhage A. A. (2013). Distinct amino acids of histone H3 control secondary metabolism in *Aspergillus nidulans*. *Appl. Environ. Microbiol.* 79 6102–6109. 10.1128/AEM.01578-13 23892751 PMC3811361

[B72] Ochi K. Hosaka T. (2013). New strategies for drug discovery: activation of silent or weakly expressed microbial gene clusters. *Appl. Microbiol. Biotechnol.* 97 87–98. 10.1007/s00253-012-4551-9 23143535 PMC3536979

[B73] Okada B. K. Seyedsayamdost M. R. (2017). Antibiotic dialogues: induction of silent biosynthetic gene clusters by exogenous small molecules. *FEMS Microbiol. Res.* 41 19–33. 10.1093/femsre/fuw035 27576366 PMC5233716

[B74] Pan R. Bai X. Chen J. Zhang H. Wang H. (2019). Exploring Structural Diversity of Microbe Secondary Metabolites Using OSMAC Strategy: a Literature Review. *Front. Microbiol*. 10:294. 10.3389/fmicb.2019.00294 30863377 PMC6399155

[B75] Perrin R. M. Fedorova N. D. D. Bok J. W. Cramer R. A. Wortman J. R. Kim H. S. (2007). Transcriptional regulation of chemical diversity in *Aspergillus fumigatus* by LaeA. *PLoS Pathog.* 3:e50. 10.1371/journal.ppat.0030050 17432932 PMC1851976

[B76] Pettit R. K. (2011). Small-molecule elicitation of microbial secondary metabolites. *Microb. Biotechnol.* 4 471–478. 10.1111/j.1751-7915.2010.00196.x 21375710 PMC3815259

[B77] Pfannenstiel B. T. Keller N. P. (2019). On top of biosynthetic gene clusters: how epigenetic machinery influences secondary metabolism in fungi. *Biotechnol. Adv*. 37:107345. 10.1016/j.biotechadv.2019.02.001 30738111 PMC6685777

[B78] Phukan H. Mitra P. K. Saikia M. (2018). Comparative study of Endophytic fungal metabolite isolated from black turmeric (*Curcuma caesia* roxb) in ROS associated *Caenorhabditis elegans* model system. *World J. Pharm. Res.* 4 79–82.

[B79] Pidroni A. Faber B. Brosch G. Bauer I. Graessle S. (2018). A Class 1 Histone Deacetylase as Major Regulator of Secondary Metabolite Production in *Aspergillus nidulans*. *Front. Microbiol.* 9:2212. 10.3389/fmicb.2018.02212 30283426 PMC6156440

[B80] Poças-Fonseca M. J. Cabral C. G. Manfrão-Netto J. (2020). Epigenetic manipulation of filamentous fungi for biotechnological applications: a systematic review. *Biotechnol. Lett.* 42 885–904. 10.1007/s10529-020-02871-8 32246346

[B81] Rana K. L. Kour D. Sheikh I. Yadav N. Yadav A. N. Kumar V. (2019). “Biodiversity of Endophytic Fungi from Diverse Niches and Their Biotechnological Applications,” in *Advances in Endophytic Fungal Research. Fungal Biology*, ed. Singh B. (Cham: Springer), 10.1007/978-3-030-03589-1_6

[B82] Rea S. Eisenhaber F. O’Carroll D. Strahl B. D. Sun Z. W. Schmid M. (2000). Regulation of chromatin structure by site-specific histone H3 methyltransferases. *Nature* 406 593–599. 10.1038/35020506 10949293

[B83] Reen J. Romano S. Dobson A. D. W. O’Gara F. (2015). The sound of Silence: activating Silent Biosynthetic Gene Clusters in Marine Microorganisms. *Mar. Drugs* 13 4754–4783. 10.3390/md13084754 26264003 PMC4557003

[B84] Reyes-Dominguez Y. Bok J. W. Berger H. Shwab E. K. Basheer A. Gallmetzer A. (2010). Heterochromatic marks are associated with the repression of secondary metabolism clusters in *Aspergillus nidulans*. *Mol. Microbiol.* 76 1376–1386. 10.1111/j.1365-2958.2010.07051.x 20132440 PMC2904488

[B85] Rodriguez R. J. White J. F. Jr. Arnold A. E. Redman R. S. (2009). Fungal endophytes: diversity and functional roles. *New Phytol.* 182 314–330. 10.1111/j.1469-8137.2009.02773.x 19236579

[B86] Romano S. Jackson S. A. Patry S. Dobson A. D. W. (2018). Extending the “One Strain Many Compounds” (OSMAC) Principle to Marine Microorganisms. *Mar. Drugs* 16:244. 10.3390/md16070244 30041461 PMC6070831

[B87] Rutledge P. J. Challis G. L. (2015). Discovery of microbial natural products by activation of silent biosynthetic gene clusters. *Nat. Rev. Microbiol.* 13 509–523. 10.1038/nrmicro3496 26119570

[B88] Ryan R. P. Germaine K. Franks A. Ryan D. J. Dowling D. N. (2008). Bacterial endophytes: recent developments and applications. *FEMS Microbiol. Lett.* 278 1–9. 10.1111/j.1574-6968.2007.00918.x 18034833

[B89] Scherlach K. Hertweck C. (2009). Triggering cryptic natural product biosynthesis in microorganisms. *Org. Biomol. Chem.* 7 1753–1760. 10.1039/b821578b 19590766

[B90] Scherlach K. Hertweck C. (2021). Mining and unearthing hidden biosynthetic potential. *Nat. Commun*. 12:3864. 10.1038/s41467-021-24133-5 34162873 PMC8222398

[B91] Shwab E. K. Bok J. W. Tribus M. Galehr J. Graessle S. Keller N. P. (2007). Histone Deacetylase Activity Regulates Chemical Diversity in *Aspergillus*. *Eukaryot. Cell* 6 1656–1664. 10.1128/EC.00186-07 17616629 PMC2043372

[B92] Shwab E. K. Keller N. P. (2008). Regulation of secondary metabolite production in filamentous ascomycetes. *Mycol. Res*. 112 225–230. 10.1016/j.mycres.2007.08.021 18280128

[B93] Smith W. L. Edlind T. D. (2002). Histone deacetylase inhibitors enhance *Candida albicans* sensitivity to azoles and related antifungals: correlation with reduction in CDR and ERG upregulation. *Antimicrob. Agents Chemother.* 46 3532–3539. 10.1128/AAC.46.11.3532-3539.2002 12384361 PMC128736

[B94] Strauss J. Reyes-Dominguez Y. (2011). Regulation of secondary metabolism by chromatin structure and epigenetic codes. *Fungal Genet. Biol.* 48 62–69. 10.1016/j.fgb.2010.07.009 20659575 PMC3935439

[B95] Strobel G. A. (2003). Endophytes as sources of bioactive compounds. *Microbes Infect.* 5 535–544. 12758283 10.1016/s1286-4579(03)00073-x

[B96] Studt L. Schmidt F. J. Jahn L. Sieber C. M. K. Connolly L. R. Niehaus E.-M. (2013). Two histone deacetylases, FfHda1 and FfHda2, are important for *Fusarium fujikuroi* secondary metabolism and virulence. *Appl. Environ. Microbiol.* 79 7719–7734. 10.1128/AEM.01557-13 24096420 PMC3837819

[B97] Sun J. Awakawa T. Noguchi H. Abe I. (2012). Induced production of mycotoxins in an endophytic fungus from the medicinal plant *Datura stramonium* L. *Bioorganic Med. Chem. Lett.* 22, 6397–6400. 10.1016/j.bmcl.2012.08.063 22967766

[B98] Sun R. Wen M. Wu L. Lan H. Yuan J. Wang S. (2021). The Fungi-specific histone Acetyltransferase Rtt109 mediates morphogenesis, Aflatoxin synthesis and pathogenicity in *Aspergillus flavus* by acetylating H3K9. *IMA Fungus* 12:9. 10.1186/s43008-021-00060-4 33823938 PMC8025522

[B99] Takahashi J. A. Gomes D. C. Lyra F. H. dos Santos G. F. (2016). “Modulation of fungal secondary metabolites biosynthesis by chemical epigenetics,” in *Fungi: Applications Management Strategies*, eds Deshmukh S. K. Misra J. K. Tewari J. P. (Boca Raton: CRC Press), 117–133.

[B100] Toghueo R. Sahal D. Boyom F. F. (2020). Recent advances in inducing endophytic fungal specialized metabolites using small molecule elicitors including epigenetic modifiers. *Phytochemistry* 174:112338. 10.1016/j.phytochem.2020.1123 32179305

[B101] Tribus M. Bauer I. Galehr J. Rieser G. Trojer P. Brosch G. (2010). A novel motif in fungal class 1 histone deacetylases is essential for growth and development of *Aspergillus*. *Mol. Biol. Cell* 21, 345–353. 10.1091/mbc.e09-08-0750 19940017 PMC2808227

[B102] Tyurin A. P. Alferova V. A. Korshun V. A. (2018). Chemical Elicitors of Antibiotic Biosynthesis in Actinomycetes. *Microorganisms* 6:52. 10.3390/microorganisms6020052 29890642 PMC6027282

[B103] VanderMolen K. M. Darveaux B. A. Chen W. L. Swanson S. M. Pearce C. J. Oberlies N. H. (2014). Epigenetic Manipulation of a Filamentous Fungus by the Proteasome-Inhibitor Bortezomib Induces the Production of an Additional Secondary Metabolite. *RSC Adv.* 4 18329–18335. 10.1039/C4RA00274A 24955237 PMC4061710

[B104] VanderMolen K. M. Raja H. A. El-Elimat T. Oberlies N. H. (2013). Evaluation of culture media for the production of secondary metabolites in a natural products screening program. *AMB Express* 3:71. 10.1186/2191-0855-3-71 24342059 PMC3917616

[B105] Vrba J. Trtkova K. Ulrichova J. (2011). HDAC inhibitors sodium butyrate and sodium valproate do not affect human ncor1 and ncor2 gene expression in HL-60 cells. *Biomed. Pap. Med. Fac. Univ. Palacky Olomouc Czech.* 155 259–262. 10.5507/bp.2011.033 22286811

[B106] Wang X. Sena Filho J. G. Hoover A. R. King J. B. Ellis T. K. Powell D. R. (2010). Chemical epigenetics alters the secondary metabolite composition of guttate excreted by an atlantic-forest-soil-derived *Penicillium citreonigrum*. *J. Nat. Prod.* 73 942–948. 10.1021/np100142h 20450206 PMC2878378

[B107] Williams R. B. Henrikson J. C. Hoover A. R. Lee A. E. Cichewicz R. H. (2008). Epigenetic remodeling of the fungal secondary metabolome. *Org. Biomol. Chem.* 6 1895–1897. 10.1039/b804701d 18480899

[B108] Wu J. S. Shi X. H. Zhang Y. H. Yu J. Y. Fu X. M. Li X. (2019). Co-cultivation With 5-Azacytidine Induced New Metabolites From the Zoanthid-Derived Fungus *Cochliobolus lunatus*. *Front. Chem.* 7:763. 10.3389/fchem.2019.00763 31781545 PMC6857680

[B109] Wu M. Y. Yu J. H. (2015). “Epigenetics of fungal secondary metabolism related genes,” in *Biosynthesis and Molecular Genetics of Fungal Secondary Metabolites*, eds Zeilinger S. Martín J. F. García-Estrada C. (New York: Springer), 29–42.

[B110] Xu F. Nazari B. Moon K. Bushin L. B. Seyedsayamdost M. R. (2017). Discovery of a Cryptic Antifungal Compound from *Streptomyces albus* J1074 Using High-Throughput Elicitor Screens. *J. Am. Chem. Soc.* 139 9203–9212. 10.1021/jacs.7b02716 28590725 PMC5617735

[B111] Yang X. L. Awakawa T. Wakimoto T. Abe I. (2013). Induced production of novel prenyldepside and coumarins in endophytic fungi *Pestalotiopsis acacia*. *Tetrahedron Lett.* 54 5814–5817.

[B112] Zhang W. Huang J. Cook D. E. (2021). Histone modification dynamics at H3K27 are associated with altered transcription of in planta induced genes in *Magnaporthe oryzae*. *PLoS Genet.* 17:e1009376. 10.1371/journal.pgen.1009376 33534835 PMC7886369

[B113] Zutz C. Bacher M. Parich A. Kluger B. Gacek-Matthews A. Schuhmacher R. (2016). Valproic Acid Induces Antimicrobial Compound Production in *Doratomyces microspores*. *Front. Microbiol.* 7:510. 10.3389/fmicb.2016.00510 27148199 PMC4829596

[B114] Zutz C. Bandian D. Neumayer B. Speringer F. Gorfer M. Wagner M. (2014). Fungi treated with small chemicals exhibit increased antimicrobial activity against facultative bacterial and yeast pathogens. *BioMed Res. Int.* 2014:540292. 10.1155/2014/540292 25121102 PMC4119895

[B115] Zutz C. Gacek A. Sulyok M. Wagner M. Strauss J. Rychli K. (2013). Small chemical chromatin effectors alter secondary metabolite production in *Aspergillus clavatus*. *Toxins* 5 1723–1741. 10.3390/toxins5101723 24105402 PMC3813908

